# Evaluation of an intervention to promote walking during the commute to work: a cluster randomised controlled trial

**DOI:** 10.1186/s12889-019-6791-4

**Published:** 2019-04-24

**Authors:** Suzanne Audrey, Harriet Fisher, Ashley Cooper, Daisy Gaunt, Kirsty Garfield, Chris Metcalfe, William Hollingworth, Fiona Gillison, Marie Gabe-Walters, Sarah Rodgers, Adrian L. Davis, Philip Insall, Sunita Procter

**Affiliations:** 1 Population Health Sciences, Bristol Medical School, University of Bristol, Bristol, England; 2 Centre for Exercise, Nutrition and Health Sciences and National Institute for Health Research Bristol Biomedical Research Centre, University Hospitals Bristol NHS Foundation Trust and University of Bristol, Bristol, England; 3 Bristol Randomised Trials Collaboration, Population Health Sciences, Bristol Medical School, University of Bristol, Bristol, England; 4 Department for Health, University of Bath, Bath, England; 5 Swansea University Medical School, Swansea, Wales; 6 Transport Research Institute, Edinburgh Napier University, Edinburgh, Scotland; 7 Insall & Coe, Bristol, England; 8 Bristol Medical School, University of Bristol, Canynge Hall, Whatley Road, Bristol, BS8 2PS England

**Keywords:** Walking, Active travel, Workplace, Behavioural intervention, Randomised controlled trial, Physical activity

## Abstract

**Background:**

Opportunities for working adults to accumulate recommended physical activity levels (at least 150 min of moderate intensity physical activity in bouts of at least 10 min throughout the week) may include the commute to work. Systematic reviews of interventions to increase active transport suggest studies have tended to be of poor quality, relying on self-report and lacking robust statistical analyses.

**Methods:**

We conducted a multi-centre parallel-arm cluster randomised controlled trial, in workplaces in south-west England and south Wales, to assess the effectiveness of a behavioural intervention to increase walking during the commute. Workplace-based Walk to Work promoters were trained to implement a 10-week intervention incorporating key behavioural change techniques: providing information; encouraging intention formation; identifying barriers and solutions; goal setting; self-monitoring; providing general encouragement; identifying social support; reviewing goals, and; relapse prevention. Physical activity outcomes were objectively measured using accelerometers and GPS receivers at baseline and 12-month follow-up. The primary outcome was daily minutes of moderate to vigorous physical activity (MVPA). Secondary outcomes included overall levels of physical activity and modal shift (from private car to walking). Cost-consequences analysis included employer, employee and health service costs and outcomes.

**Results:**

Six hundred fifty-four participants were recruited across 87 workplaces: 10 micro (5–9 employees); 35 small (10–49); 22 medium (50–250); 20 large (250+). The majority of participants lived more than two kilometres from their place of work (89%) and travelled to work by car (65%). At 12-month follow-up, 84 workplaces (41 intervention, 43 control) and 477 employees (73% of those originally recruited) took part in data collection activities. There was no evidence of an intervention effect on MVPA or overall physical activity at 12-month follow-up. The intervention cost on average £181.97 per workplace and £24.19 per participating employee.

**Conclusions:**

The intervention, focusing primarily on individual behaviour change, was insufficient to change travel behaviour. Our findings contribute to the argument that attention should be directed towards a whole systems approach, focusing on interactions between the correlates of travel behaviour.

**Trial registration:**

ISRCTN15009100. Prospectively registered. (Date assigned: 10/12/2014).

## Background

### Physical inactivity and health

Increasing physical activity levels, especially among the least active, is an important aim of public health policy. Lack of physical activity is associated with an increased risk of chronic diseases such as type 2 diabetes, heart disease and some cancers [1, 2]. It is recommended that adults achieve at least 150 min of moderate physical activity during the week [3, 4]. However, many adults in high income countries, including the United Kingdom (UK), do not achieve this [5]. ‘Moderate intensity’ physical activity can be achieved by walking at a speed of five kilometres per hour (approximately three miles per hour) [6]. A systematic review found evidence that walking interventions improve CVD risk factors for previously inactive healthy adults [7]. Adults who commute to work by active and public modes of transport have been shown to have significantly lower body mass index (BMI) and percentage body fat than their counterparts using private cars [8].

### Walking as active travel

In the UK, opportunities exist to increase walking by replacing short car journeys: the National Travel Survey 2016 showed 24.5% of all car journeys were shorter than two miles (3.2 km), and 13% of journeys less than one mile (1.6 km) were made by car [9]. An opportunity for working adults to accumulate the recommended activity levels may be through the daily commute.

Systematic reviews have examined the effectiveness of interventions to promote physical activity [10], walking and cycling as an alternative to car use [11], and workplace physical activity [12]. None have focussed specifically on interventions that promote walking during the commute. Furthermore, few studies have examined how workplace physical activity interventions are influenced by size and type of workplace or characteristics of employees [1]. A systematic review of the effectiveness of interventions to change from car to active transport concluded that intervention characteristics were poorly described, and the studies were predominantly of poor quality and lacked robust statistical analyses [13].

### Measuring physical activity

A systematic review comparing measures for assessing adult physical activity found self-report measures were higher than objective measures in some cases and lower in others [14]. This casts doubt on the reliability of self-report, and impedes correction for measurement error. However, self-reported measures of physical activity remain common and few studies have objectively measured the contribution of walking, particularly walking to work, to adult physical activity levels [12, 15].

### Costs and benefits of walking as active travel

Although studies have shown health benefits from active commuting, there are very few studies have assessed the cost effectiveness of active travel interventions. A systematic review of interventions to promote walking included 19 randomised controlled trials and 29 non-randomised controlled studies but only six studies included even rudimentary economic evaluation [16].

### Aim and objectives of the current study

The overall aim of this study is to evaluate the effectiveness of a workplace-based intervention to increase walking during the commute. The objectives were: to provide objective evidence of participating employees’ moderate to vigorous physical activity (MVPA), overall physical activity, sedentary time, MVPA associated with the commute, and mode of travel. A further objective is to provide evidence on the cost and economic benefits of the intervention to employers, employees and society (commuting costs, health service use, lost productivity, well-being).

## Methods

The study builds on findings from the Walk to Work feasibility study (NIHR-PHR project 10/3001/04) [17]. The methods section of this paper draws on the trial protocol and the statistical and health economics analysis plan that are described in detail elsewhere [18] (http://research-information.bristol.ac.uk/files/118260521/TtW_SHEAP_V.1.1_signed.pdf), and summarised in Audrey and Fisher, 2019 [19].

### Trial design

The study is a multi-centre parallel arm cluster randomised controlled trial. A cluster trial design, with the workplace as the unit of randomisation, was chosen because of the potential for contamination between individuals in the same workplace. The study included health economic costs and outcomes, and process evaluation.

### Sample size

Using findings from the feasibility study [17], the sample size for this full-scale trial was based on an average cluster size of eight people, an intra-cluster correlation coefficinet (ICC) of 0.15, and 25% attrition. We calculated 678 employees were required across 84 workplaces (42 intervention, 42 control) to detect a 15% difference in MVPA (equal to a difference of 0.36 standard deviations) with 80% power at the 5% significance level.

### Recruitment and eligibility

Information about the study was sent to workplaces in seven urban areas in south-west of England and south Wales identified through business directories and local authority lists of employers. The information included a short form to complete to express interest in taking part in the study. Very small workplaces (< 5 employees) were not recruited. Because of the need for a 12-month follow-up, workplaces employing predominantly casual staff on short-term contracts, and workplaces that were intending to relocate or downsize were not eligible.

Employees whose job required them to drive to work, or who already always walked or cycled to work, were ineligible for the study, because the intervention was aimed at increasing active travel during the commute. Employees who intended to leave the workplace within the next 12 months were also ineligible because of the need for the 12-month follow-up.

### Consent procedures

Eligible employers who expressed interest in the study were sent further information and a workplace consent form. When consent was received at workplace level, employers were asked to distribute study information leaflets and consent forms to eligible employees. Consent was obtained before baseline data were collected and workplaces randomised to the intervention or control arm.

In the intervention arm, potential Walk to Work promoters were given information about the role and consent was given before they were trained to deliver the intervention. The University of Bristol Faculty of Health Sciences Research Ethics Committee gave ethical approval for the study.

### Physical activity measures and outcomes

Accelerometers (Actigraph GT1M) were used to measure participants’ physical, using validated accelerometer thresholds to compute MVPA and sedentary time [20]. Decisions and outcomes for analysis of accelerometer data are listed in Table 1. Participants were also asked to wear GPS receivers (QStarz BT1000X) set to record positional data every 10 s. GPS and accelerometer data were time-matched and visualised in a Geographic Information System (GIS; ArcMap v10.2.2). Journeys were manually identified and the data segmented to calculate duration and MVPA accrued.

**Table 1:** Accelerometry: decisions and outcomes

Initialising	Accelerometers initialised to start recording on day after distribution and to store data for 7 days including a weekend.
Data collection points	Baseline, 1-year follow-up
Protocol	Single Actigraph GT3X+ monitor, worn around the waist over the same hip during waking hours (except when swimming/bathing/showering).
Wear time	Waking hours (usually 6.00 am – midnight but this will be modified, for example, for shift workers)
Valid length of day	≥10 h (600 min)
Days required	3 days
Epoch length	10 s
Zero counts	Bouts of 60 min of continuous/consecutive zero counts excluded
Spurious data	> 15,000 cpm
Missing data	No imputation
Activity cut-points	Sedentary < 100 cpm; MVPA ≥1952 cpm [19]
Outcomes	i) Moderate to vigorous physical activity (MVPA) ii) Sedentary time iii) Overall physical activity, mean counts per minutes (cpm)

The primary outcome was daily minutes of MVPA. Secondary outcomes were: overall levels of physical activity (average counts per minute (cpm) of all valid days, calculated from total number of counts divided by the time the accelerometer was worn); daily minutes of sedentary time; daily minutes of MVPA during the commute, and; modal shift (number of journeys, over the previous five working days, when walking was the major commuting mode). Outcomes were measured at baseline and 12-month follow-up.

### Health economic costs and outcomes

The costs of the intervention included Walk to Work promoter training (trainer time and travel costs, and promoter time) and intervention resources (e.g. booklets, pedometers). Promoter time was valued by dividing the upper quartile weekly earnings reported in the annual survey of hours and earnings by the median number of hours worked per week [21]. University of Bristol pay scales were used to value trainer time, and trainer travel costs were either self-reported or estimated using a cost per mile of 59.9 pence for car travel [22]. Intervention resources were valued at purchase cost.

At baseline and 12-month follow-up health care resource use was measured and valued using 2016 unit costs [23–25]. Self-reported workplace productivity was measured at baseline, post-intervention (3-month follow-up), and 12-month follow-up using a 10-point scale (ranging from health problems “had no effect on my work” to “completely prevented me from working”) and as “days of work missed because of your health problems” [26]. This was valued using median weekly earnings [21].

A measure of wellbeing, ICECAP-A [27], was collected at baseline, 3-month follow-up and 12-month follow-up. ICECAP-A is scored on a scale from zero to one, with higher scores reflecting better wellbeing. A repeated measures linear regression analysis was undertaken to examine the intervention effect on productivity, commute costs and wellbeing with time point, baseline measure of the dependent variable, workplace type, workplace size and workplace location as covariates, and including workplace as a random effect.

At baseline and 12-month follow-up, participants were asked to record commute costs and time in a weekly travel diary. If participants included time spent on other activities (e.g. shopping) as part of their commute, the mean commute time for the given transport mode was used where possible. Commute costs were self-reported, and time spent travelling by car was valued using an average speed of 23.43 mph [8] and a cost per mile of 59.9 pence [22].

### Randomisation procedures

Randomisation, to receive the Walk to Work intervention or continue with usual practice, took place at workplace level. Workplaces were matched in pairs (or in some cases as triples where this provided a better fit) based on location, size (micro, small, medium and large), and economic activity (using United Kingdom standard industrial classification of economic activities (UK-SIC) categories) (https://www.gov.uk/government/publications/standard-industrial-classification-of-economic-activities-sic). Assignment of workplaces was undertaken by a Bristol Randomised Trials Collaboration statistician who was not involved in recruiting workplaces, using random numbers generated by Stata Version 14 (College Station, Texas, US). One workplace from a matched set was randomised to the control group and one (or two in a triple) to receive the intervention. The intervention activities meant it was not possible to blind participants following randomisation.

### The walk to work intervention

Behavioural change techniques (BCTs) have been defined as the ‘active ingredients’ within interventions designed to change individual behaviour [28]. The Walk to Work intervention included the following BCTs: providing information (about the benefits of walking to work); encouraging intention formation; identifying barriers and solutions; goal setting; self-monitoring (with travel diaries and optional pedometers); providing general encouragement; identifying social support; reviewing goals, and; relapse prevention [28].

There were three main stages of the 10-week intervention: identification and training of workplace Walk to Work promoters; initial contact between the Walk to Work promoters and participating employees, including the distribution and discussion of intervention materials, and: three additional contacts during the following 10 weeks to provide encouragement for participating employees and Walk to Work promoters. Ten weeks is considered a suitable length of time to enable a change of behaviour to become a habit [29].

#### Walk to work promoter recruitment and training

Following randomisation, workplaces receiving the intervention identified a Walk to Work promoter, for example an employee already tasked with developing and implementing a travel plan or a volunteer who was keen to promote walking to work. At some workplace, the employer undertook the role. Potential Walk to Work promoters were provided with an information leaflet explaining the role and a consent form. Written consent was provided before the Walk to Work promoters received the training and undertook the role.

The research team delivered a training session, lasting approximately 1 h, to the Walk to Work promoters at their workplace and at a time and place to suit their needs. A DVD, summarising the training was developed by the research team and given to the promoters for future reference. The training included: information about the health, social, economic and environmental benefits of walking; using behavioural change techniques to promote increased walking, either the whole route or as part of a mixed-mode journey; providing support and encouragement to participating employees, and; accessing relevant websites and resources for additional information and resources. Walk to Work promoters were given booklets, also developed by the research team, to assist them in the role. The content of these booklets was discussed during the training session.

Walk to Work promoters were given the names of the employees in their workplace who had consented to take part in the study. They were asked to distribute the Walk to Work booklets and optional pedometers, and to encourage behavioural change during four contacts over the 10-week intervention period. Contacts between the Walk to Work promoters and participating employees could be organised to suit the circumstances of workplaces and their employees e.g. in groups or on an individual basis, in person or by email. The Walk to Work promoter’s booklet contained guidance about providing support and encouragement to participating employees and optional diary pages to record their activities.

Walk to Work promoters were sent three newsletters from the research team during the intervention period. These were supplemented by newsletters to pass on to participants focusing on specific BCTs and providing additional information about the benefits of walking. The newsletters were provided by email and/or paper copy to suit the workplace.

#### Participant booklets and newsletters

The Walk to Work promoters were given the names of all employees in their workplaces who were participating in the study and were asked to provide each of them with a participant booklet. These booklets provided information and guidance relating to the BCTs that comprised the behavioural intervention. To encourage intention formation, the booklets began by considering the benefits of increasing walking during the commute. The focus then moved to identifying personal benefits, barriers and solutions, and goal setting. Self-monitoring was encouraged through the use diary pages at the back of the participant’s booklet or the use of optional pedometers to record daily steps. Participants were prompted to seek encouragement and social support during their attempts to increase walking during the commute.

Three newsletters were sent to the Walk to Work promoters for circulation to participating employees during the following 10 weeks. These provided additional information, to stimulate continued interest in the benefits of walking, as well as continuing the focus on key BCTs: highlighting social support around Week 3, reviewing goals at Week 5, and following up participants to support the maintenance of behavioural change (relapse prevention) around Week 7.

#### Information and ideas for employers

An employer pack was provided to all workplaces in the intervention arm of the study. This comprised a letter outlining the intervention, the booklets being used by the Walk to Work promoters and participants, additional booklets specifically designed for employers, and poster templates that could be modified for displayed in the workplace. The employer’s booklet contained ideas for promoting walking to work including: providing information about walking distances to train and bus stops; providing lockers or improved cloakroom facilities; giving financial assistance for public transport season tickets or walking clothes/shoes; offering free incentive items for those who switch to walking e.g. umbrellas, rucksacks, or breakfast vouchers; supporting competitions and challenges for those who enjoy taking part in such activities. Employers were encouraged to record any support they had provided for the intervention, together with associated costs, in a section at the back of their booklet.

### Process evaluation

A mixed-method process evaluation, to be reported in detail elsewhere, included interviews and survey questions to explore the context, delivery of, and response to the Walk to Work intervention. The post-intervention questionnaire (3-month follow-up), for both intervention and control groups, included the question “In the last 2-3 months, has anyone in your workplace tried to encourage you to change the way you travel to or from work?”

### Statistical methods

An analysis plan was made publicly available before outcome data were released to the statistical team (http://research-information.bristol.ac.uk/files/118260521/TtW_SHEAP_V.1.1_signed.pdf). Study participants who provided a measurement of the primary outcome were included in the primary analysis, comparing intervention with usual practice as allocated. The treatment effect was estimated as a mean difference using multivariable linear regression, including treatment arm, baseline MVPA, workplace size, location, and type of business as covariates, and the workplaces as a normally distributed random effect (to take account of clustering). This approach was adapted to the secondary outcome measures, with a zero-inflated negative binomial regression model, with robust standard errors, estimating treatment effect on the modal shift measure (number of journeys when walking was the major mode of travel).

Pre-planned sensitivity analyses assessed the impact on: the primary analysis of any imbalance in baseline covariates; any non-normality in the distribution of the primary outcome, and; different quality assurance thresholds for accelerometer data. The latter analysis included more participants in the primary analysis and so explored the influence of missing values. Subgroup analyses of the primary outcome measure explored whether age at baseline (above/below the median), sex (male/female), or household income (above/below £30,000) modified the intervention effect; these analyses proceeded by adding interaction terms to the regression models used in the primary analysis.

## Results

### Recruitment and retention

**Fig. 1:**
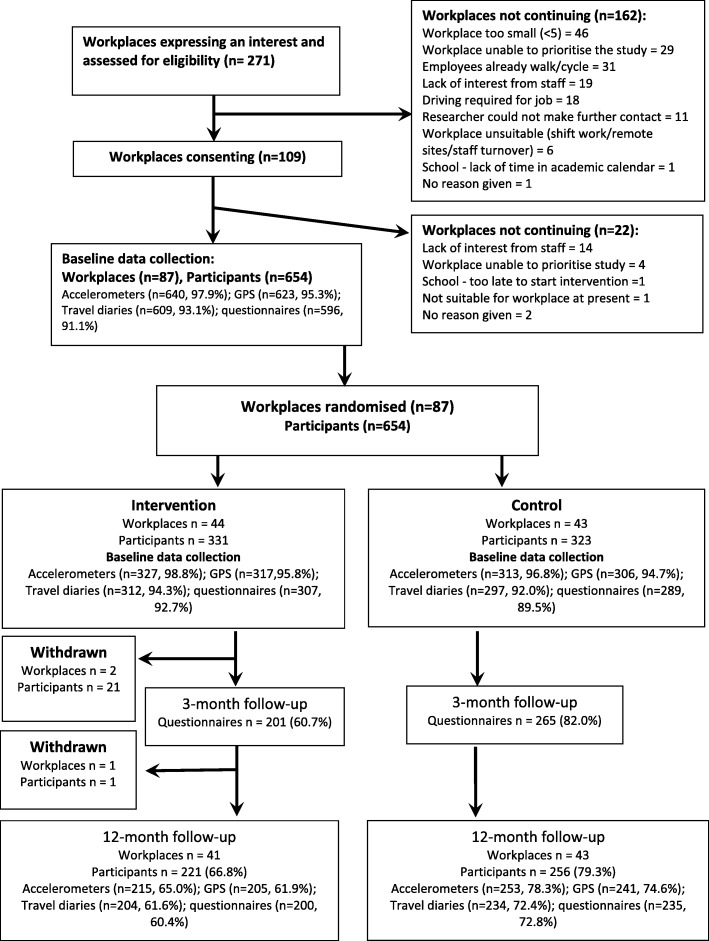
Flow of workplaces and participants throughout the study

Recruitment took place across seven urban areas in south-west England and south Wales during May–July 2015 and March–May 2016. We sent out information about the study using available lists of employers for each area. We received 271 expressions of interest and, after screening for eligibility and giving further information about the study, we recruited 87 workplaces (Fig. 1): 10 micro (5–9 employees); 35 small (10–49); 22 medium (50–250); 20 large (250+).

Following baseline data collection, 44 workplaces (331 participants) were randomised to receive the intervention, and 43 workplaces (323 participants) to the control arm (Fig. 1). Two workplaces (21 participants) withdrew during the 10-week intervention period: one relocated overseas, and in the other workplace the main contact indicated they were too busy to continue with the study. A further intervention workplace (1 participant) withdrew at the follow-up data collection because of lack of interest amongst colleagues. At 12-month follow-up 84 workplaces (41 intervention, 43 control) and 477 employees (73% of those originally recruited to the study) took part in data collection activities, with response rates higher in the control arm (Fig. 1).

Tables 2 and 3 show the baseline characteristics of workplaces and participants in the study and illustrate the balance between intervention and control arms following randomisation. We recruited workplaces with a range of different sizes and industrial classifications, in which most employees travelled to work by car. Within these workplaces study participants were predominantly White British (90%), qualified to degree level or above (60%), in sedentary occupations (77%) and lived more than two kilometres from their place of work (89%).

**Table 2:** Baseline characteristics of participating workplaces (*n* = 87)

	Intervention (*n* = 44)	Control (n = 43)
	n (%)	n (%)
Location		
Swansea (including Newport and Neath Port Talbot)	13 (30%)	13 (30%)
Bath (including Swindon)	8 (18%)	6 (14%)
Bristol (including South Gloucestershire)	23 (52%)	24 (56%)
Size of business		
Micro (5–9 employees)	4 (9%)	6 (14%)
Small (10–49 employees)	21 (48%)	14 (33%)
Medium (50–250 employees)	9 (20%)	13 (30%)
Large (250+ employees)	10 (23%)	10 (23%)
Most often used method of travel to work by employees		
Car or motorised transport	32 (73%)	31 (72%)
Public transport	1 (2%)	1 (2%)
Walk or cycle	1 (2%)	0 (0%)
Unknown	10 (23%)	11 (26%)
Proportion of employees that walk or cycle all the way to work		
None or hardly any	13 (30%)	12 (28%)
Less than half	23 (52%)	21 (49%)
Most	1 (2%)	0 (0%)
All	1 (2%)	0 (0%)
Unknown	6 (14%)	10 (23%)
UK Standard Industrial Classification (SIC) Categories 2007		
C: Manufacturing	4 (9%)	2 (5%)
D: Electricity, gas, steam & air conditioning supply	0 (0%)	1 (2%)
F: Construction	1 (2%)	0 (0%)
G: Wholesale & retail trade; repair of motor vehicles & motor cycles	4 (9%)	2 (5%)
H: Transport & storage	0 (0%)	1 (2%)
K: Financial & insurance activities	2 (5%)	2 (5%)
M: Professional, scientific & technical activities	10 (23%)	11 (26%)
N: Administrative & support service activities	5 (11%)	3 (7%)
O: Public administration & defence; compulsory social security	4 (9%)	4 (9%)
P: Education	5 (11%)	6 (14%)
Q: Human health & social work activities	6 (14%)	5 (12%)
R: Arts, entertainment & recreation	1 (2%)	4 (9%)
S: Other service activities	2 (5%)	2 (5%)

**Table 3:** Baseline characteristics of participants (*n* = 654)

	Intervention		Control	
	n	n (%)	n	n (%)
Participant Demographics				
Total participants	331		323	
Gender: Male	331	143 (43%)	323	140 (43%)
Age (years)	321	41.2 (11.4)	314	42.0 (11.3)
BMI:				
Underweight and normal	331	149 (45%)	323	144 (45%)
Overweight		99 (30%)		92 (28%)
Obese		53 (16%)		52 (16%)
Missing		30 (9%)		35 (11%)
Household income:				
Up to £10,000	313	1 (< 1%)	305	3 (1%)
£10,001 - £20,000		14 (4%)		25 (8%)
£20,001 - £30,000		39 (12%)		39 (13%)
£30,001 - £40,000		51 (16%)		49 (16%)
£40,001 - £50,000		67 (21%)		53 (17%)
More than £50,000		118 (38%)		117 (38%)
Don’t know		23 (7%)		19 (6%)
Ethnicity:				
White British	317	288 (91%)	310	279 (90%)
White other		15 (5%)		14 (5%)
Mixed ethnic group		4 (1%)		3 (1%)
Asian or British Asian		3 (1%)		6 (2%)
Black or Black British		7 (2%)		5 (2%)
Chinese		0 (0%)		3 (1%)
Education:				
Higher degree, degree or equivalent	315	195 (62%)	309	182 (59%)
A-levels or equivalent		74 (23%)		79 (26%)
GCSEs or equivalent		41 (13%)		43 (14%)
No formal qualifications		5 (2%)		5 (2%)
Current method of travel to work (by journeys)				
Car	327	217 (66%)	313	205 (65%)
Public transport		44 (13%)		32 (10%)
Walk		32 (10%)		42 (13%)
Cycle		34 (10%)		34 (11%)
Distance between workplace and home (km)				
2 km or less	319	35 (11%)	307	30 (10%)
Over 2 km		280 (88%)		277 (90%)
Current occupation				
Sedentary	315	239 (76%)	299	237 (79%)
Standing		60 (19%)		42 (14%)
Manual		15 (5%)		20 (7%)
Heavy manual work		1 (< 1%)		0 (0%)

### Intervention delivery

The intervention was delivered during spring and summer when the weather was more conducive to active travel: the aim was to encourage walking as a habit that would then be carried forward into the autumn and winter months. All workplaces randomised to the intervention arm (*n* = 43) received the Walk to Work promoter training session and relevant booklets and resources. Following the loss of two workplaces, the Walk to Work promoters in 41 workplaces received four newsletters over the 10-week intervention period to disseminate to participating employees. In the post-intervention questionnaires (at 3-month follow-up), 33% (*n* = 66 of 201) of respondents in the intervention arm indicated “Yes” to the question “In the last 2-3 months, has anyone in your workplace tried to encourage you to change the way you travel to or from work?” compared to 13% (*n* = 35 of 265) in the control arm.

### Adverse events

No accidents or adverse events relating to the intervention were reported during the study.

### Outcomes and estimation

At the 12-month assessment point, no increase in MVPA was observed in either the intervention or control group, and hence there was no evidence of an effect of the Walk to Work intervention on the primary measure of outcome (*p* = 0.92, Table 4). The ICC for the primary outcome was estimated at 0.020 (95% confidence intervals (CI) 0.001 to 0.292). Including participants with at least 1 day of valid accelerometer data in a sensitivity analysis supported the same conclusion (Table 4).

**Table 4:** Summary statistics and intervention effect estimates (intervention minus control) for primary and secondary measures of physical activity

Outcome	Intervention mean (SD) N		Controlmean (SD) N		Adjusted difference in means^a^ (95% CI)	*P*-value^a^
	Baseline	Follow-up	Baseline	Follow-up		
Primary						
i) Daily minutes of moderate to vigorous physical activity (MVPA)	55.0 (24.9) 142	53.3 (23.7) 142	57.7 (37.0) 180	53.9 (27.6) 180	0.3 (−5.3, 5.9)	0.917
Secondary						
ii) Overall physical activity (counts per minute)	390.5 (144.2) 142	387.6 (148.5) 142	417.1 (267.4) 180	392.7 (168.8) 180	3.5 (−30.3, 37.4)	0.838
iii) Sedentary time (minutes per day)	585.5 (63.3) 141	580.0 (97.0) 141	581.9 (80.1) 178	585.4 (108.6) 178	1.0 (−11.7, 13.6)	0.882
iv) Daily minutes of MVPA during commute	13.9 (14.1) 183	13.8 (14.0) 183	13.3 (15.1) 213	16.2 (19.0) 213	−3.1 (−6.0, −0.2)	0.036
Sensitivity analyses of primary outcome						
Daily minutes of MVPA: 1 day of valid data	52.6 (25.0) 189	51.1 (23.7) 189	55.5 (35.1) 217	52.6 (28.1) 217	−0.4 (−5.3, 4.5)	0.876

^a^Multi-level mixed effect linear regression model adjusted for size, location and type of business, baseline outcome, accelerometer wear-time at follow-up (for outcomes i and iii) and workplace as a random effect

The unadjusted means of the primary outcome, daily minutes of MVPA, and the secondary outcome, overall physical activity, decreased in both intervention and control groups over the 12-month follow-up (Table 4). There was no evidence of an effect of the intervention on these measures for any of the adjusted analyses. The unadjusted means of sedentary time increased over the 12-month follow-up in the control arm but reduced in the intervention arm, again with no evidence of an intervention effect. However, daily minutes of MVPA during the commute increased in the control arm but reduced in the intervention arm with evidence of a differential effect after adjustment for covariates (*p* = 0.036). For both intervention and control groups, at all assessment points, the median number of journeys walked to work was 0 (IQR 0 to 0), with no evidence of an effect of the intervention on this measure (*p* = 0.395).

There was no evidence that the effect of the intervention differed between different age groups, males and females, or participants differing in household income (Table 5).

**Table 5:** Subgroup analyses of primary outcome

Outcome	Intervention mean (SD) n		Control mean (SD) n		Adjusted difference in means between control and intervention within subgroup* (95% CI)	Interaction test P-value*
	Baseline	Follow-up	Baseline	Follow-up		
Daily minutes of moderate to vigorous physical activity (MVPA)						
Age (<median)	55.2 (23.2) 61	56.3 (24.7) 61	63.2 (46.7) 77	60.6 (26.8) 77	−1.9 (−9.8, 6.0)	
Age (≥median)	54.9 (26.3) 81	51.0 (22.8) 81	53.6 (27.2) 103	48.9 (27.2) 103	1.6 (−5.5,8.6)	0.496
Sex (Male)	58.1 (24.8) 57	56.4 (23.7) 57	61.8 (50.1) 71	56.0 (31.3) 71	1.5 (−6.8, 9.9)	
Sex (Female)	53.0 (24.9) 85	51.1 (23.6) 85	55.0 (24.9) 109	52.6 (25.0) 109	−0.8 (−7.7, 6.2)	0.664
Household income (below £30,000 or missing)	52.4 (24.2) 32	54.4 (23.2) 32	54.6 (25.5) 41	53.8 (24.6) 41	2.6 (−8.3, 13.5)	
Household income (above £30,000)	55.8 (25.2) 110	52.9 (24.0) 110	58.6 (39.8) 139	54.0 (28.5) 139	−0.4 (−6.6, 5.8)	0.628
Distance from work (2 km or less)	58.7 (26.3) 20	57.2 (25.4) 20	59.9 (22.7) 12	64.5 (34.0) 12	−6.8 (−23.2, 9.7)	
Distance from work (more than 2 km)	54.7 (24.7) 121	52.9 (23.4) 121	57.3 (38.0) 164	53.1 (27.1) 164	0.4 (−5.5, 6.3)	0.419

*Multi-level mixed effect linear regression model adjusted for size, location and type of business, baseline outcome, accelerometer wear-time at follow-up and workplace as a random effect

### Economic evaluation

The intervention cost on average £181.97 per workplace and £24.19 per participating employee (Table 6). There was no clear association between workplace size and cost per employee.

**Table 6:** Average costs of intervention delivery and promoter training, by workplace size

Workplace size^a^	Intervention materials costs (£)^b^	Promoter training		Total cost per workplace (£)	Cost per employee (£)
		Training delivery cost (£)^c^	Promoter cost(£)^d^		
Micro	31.58	130.50	20.69	182.77	43.01
Small	42.78	97.86	19.05	159.68	25.60
Medium	44.55	134.19	29.12	207.86	34.01
Large	73.26	107.08	24.83	205.16	16.03
All	49.05	110.35	22.57	181.97	24.19

^a^Size: micro 5–9 employees; small 10–49; medium 50–250; large 250+,

^b^Intervention materials costs include booklets, folders, newsletters, pedometers, posters, DVDs

^c^Trainer costs include trainer time and travel,,,

^d^Promoter costs include promoter time spent at training,,

Health service costs were similar between arms at 12-month follow-up. Estimated productivity lost from self-rated productivity at work scores suggest that participants in the control arm had more lost productivity due to ill health with an adjusted difference in wages of -£231.35 (95% CI: -£424.77 to -£37.92). However, productivity lost due to days off work did not suggest a difference (Table 7). At follow-up, on average the intervention group spent less total time commuting than the control group (adjusted incremental difference: − 9.17 min [95% CI: -18.05 to − 0.28 min]) however there was no evidence of a difference in commuting costs between arms (Table 7). ICECAP-A results (Table 7) suggest that intervention participants had marginally higher quality-of-life over follow-up than control participants [0.018, 95% CI: 0.000 to 0.036].

**Table 7:** Lost earnings, quality of life and commute costs by intervention arm

	Intervention mean (SD) n		Control mean (SD) n		Adjusted difference in means (95% CI)^b^	P-value^b^
	Post-intervention	Follow-up	Post-intervention	Follow-up		
Productivity						
Self-assessed productivity	2.230	2.262	2.563	2.853	−0.406	0.019
	(1.872)	(1.827)	(2.173)	(2.450)	(−0.744 to −0.067)	
	200	195	263	231		
Self-reported days of work missed	1.005	2.013	1.441	1.709	−0.142	0.733
	(2.795)	(6.111)	(5.125)	(4.579)	(−0.961 to 0.677)	
	199	194	262	232		
Lost earnings						
Based on self-assessed productivity	£701.32	£719.30	£891.04	£1056.44	-£231.35	0.019
	(£1067.41)	(£1041.80)	(£1238.79)	(£1397.15)	(−£424.77 to -£37.92)	
	200	195	263	231		
Based on self-reported days of work missed	£88.16	£176.57	£126.39	£149.92	-£12.50	0.733
	(£245.15)	(£536.05)	(£449.57)	(£401.70)	(−£84.34 to £59.33)	
	199	194	262	232		
Quality of life						
ICECAP-A	0.852	0.840	0.825	0.823	0.018	0.056
	(0.136)	(0.134)	(0.143)	(0.152)	(0.000 to 0.036)	
	197	196	264	228		
Commuting to work						
Commute costs^a^		£9.32		£10.99	-£1.15	0.245
		(£7.67)		(£12.19)	(−£3.10 to £0.79)	
		140		131		

^a^Commute costs collected at baseline and 12 months follow-up only

^b^Based on a repeated measures analysis that was adjusted for time point as a categorical variable, baseline value, workplace size, workplace location, workplace type and workplace as a random effect

## Discussion

We believe our study is the first cluster randomised controlled trial using GPS receivers and accelerometers to measure the effectiveness of an intervention to increase walking during the journey to and from work. We found no effect at 12-month follow-up on participants’ MVPA, overall physical activity or travel mode.

### Strengths and limitations

The use of objective measures, and a 12-month follow-up period, contribute valuable evidence for those who have called for greater rigour in assessing the effectiveness of physical activity interventions [12, 30], and interventions aiming to change the travel mode of commuters [13]. The study included workplaces in geographically distinct areas and of different sizes and industrial classifications which might add to its generalisability. However, it should be noted that participants were predominantly White, educated to degree level, and with a household income above the national average.

The intervention design involved recruiting and training Walk to Work promoters and providing them with materials and some follow-up support to encourage fellow employees to increase walking during the commute. Our aim was to assess whether this model of intervention was effective and cost effective. In terms of fidelity, the promoters all received the Walk to Work training session, the DVD and the relevant resources, and four prompts during the intervention period. However, it was not possible to directly measure the fidelity of the intervention as delivered by the Walk to Work promoters: they were encouraged to deliver the intervention to colleagues in a way that suited their workplace routines. We were not able to directly observe, for example, conversations promoters had with employees. This is an inevitable element of interventions of this kind and more accurately equates with the ‘real world’ than an intervention that is highly monitored throughout with the potential to increasing Hawthorne effects (through which the behaviour of participants is changed as a result of being observed in the research context). This model was relatively cheap to deliver, as shown by the economic evaluation, but left room for variation in fidelity and reach within the workplace. There is some preliminary evidence to suggest participants in the intervention arm were encouraged to change their travel behaviour, but more detailed analysis of process evaluation data is required to offer further insights into the delivery of the intervention.

We chose a high standard of compliance in relation to physical activity measurement (at least 3 days of at least 10 h per day at 12-month follow-up). In the intervention group, 142/331 (43%) provided a measure of the primary outcome, and in the control group 180/323 (56%) provided that measure. Whilst this response rate is clearly a limitation, we do not believe the missing measurements are causing the study results to be misleading. Measuring the primary outcome for all participants who provided 1 day or more of accelerometer data provided an outcome measure for 189/331 (57%) in the intervention group and 217/323 (67%) control; repeating the analysis with these data led to the same conclusion of no effect of the intervention.

There were several challenges in interpreting data provided by participants for the economic evaluation. Missing data was a problem, especially for commute costs where we report follow-up costs on 42% of intervention and 41% of control participants. This was due to participants needing to have recorded complete time and costs for each day they worked at baseline and follow-up. Participants were asked to report details of bus and train passes at the start of the diary, and only report separate daily costs of this mode when not using passes, but some participants reported the cost in both. Where participants did not record the reason for the travel cost, the costs were included with the possibility of double-counting costs already reported elsewhere.

### Costs and benefits

The intervention was relatively inexpensive to implement and fairly ‘light touch’ for employers to adopt. There was weak evidence that self-rated productivity and well-being scores were better in the intervention arm over the 12-month follow-up period. However, the lack of improvement in MVPA or active commuting, and the higher loss to follow-up in the intervention arm, caution against over-interpreting these findings.

### Generalisability

We recruited a range of workplaces of different categories and sizes from different urban locations. However, although the target for workplace recruitment was achieved, this was after a large mailout to workplaces across seven urban areas. Furthermore, there was a relatively low cluster size, even within larger workplaces. This suggests workplace-based interventions focusing on changing travel mode may be of greater interest to motivated subgroups of employers and employees [11]. Issues of context and reach will be explored further through the process evaluation.

### Factors associated with active commuting

The baseline data for this study, published in detail elsewhere [31], indicated the amount of daily MVPA accumulated during the commute was much lower for car users (7.3 min ± standard deviation 7.6) than for walkers (34.3 ± 18.6) and those who used public transport (25.7 ± 14.0). Analyses of combined GPS and accelerometer data showed those whose commute included at least 10 min of walking were more likely to have a shorter commuting distance (*p* < 0.001). Not having access to a car (p < 0.001) and lack of free workplace parking (*p* < 0.01) were both associated with walking to work and public transport use [29]. These findings may help to explain why the intervention was not effective in changing travel mode for the commute to work.

Other studies based in the UK have also found that a short distance to work [32–34], and no workplace parking [35, 36], supported walking as a mode of transport. In our study, most participants travelled further than two kilometres (*n* = 555, 84.8%) between their home and workplace. Consequently, for many of them, it may not have been feasible to adopt walking as their main mode of travel. However, combining public transport with walking may be possible for commuters with longer journeys. In the UK, a study involving 20 workplaces found that restrictions on parking (for example, by reducing the number of spaces available or introducing parking charges) together with financial assistance for public transport, contributed to a reduction in car use [37]. Other studies have suggested the availability of car parking, or the quality of commuting routes and infrastructure may be influential in changing travel mode [38, 39]. Furthermore, interventions are more effective when they coincide with naturally occurring disruption in travel habits [40], such as moving house, changing employment or reductions in workplace parking, suggesting that tailoring the timing or target group of travel-mode interventions according to the wider context could be important to its impact. A study examining individual, employment and psychosocial factors influencing walking to work found walkers were younger (< 30 years), did not have a car or free car parking at work, were more confident about including walking in their commute, and had support from colleagues [41]. Participants were less likely to walk during the commute if they perceived they lived too far away, felt walking was less convenient than car-use, needed a car for work, or had always travelled the same way.

## Conclusion

Public health researchers continue to be concerned that the adult population in high income countries does not undertake sufficient physical activity, with serious implications for health. Walking during the journey to and from work offers an opportunity to build physical activity into the daily routine. However, our results suggest an intervention focusing on the individual as the primary target for behaviour change, is not sufficient to change travel behaviour: future interventions and research may be better directed towards the wider determinants of health and a whole systems approach that focuses on interactions between the correlates of travel behaviour.

## Abbreviations

BCT: Behavioural change techniques
BMI: Body mass index
CI: Confidence intervals
CPM: Counts per minute
DVD: Digital versatile disc
GCSE: General certificate of secondary education
GIS: Geographic information system
GPS: Global positioning system
ICC: Intra-cluster correlation coefficient
ICECAP-A: ICEpop CAPability measure for Adults
MVPA: Moderate to vigorous physical activity
UK: United Kingdom
